# Blood flow influences vascular growth during tumour angiogenesis

**DOI:** 10.1038/sj.bjc.6690125

**Published:** 1999-02

**Authors:** R Nasu, H Kimura, K Akagi, T Murata, Y Tanaka

**Affiliations:** Department of Radiology, Kansai Medical University, 10–15, Fumizono-cho, Fumizono-cho, Osaka, 570, Japan

**Keywords:** neovascularization, experimental tumour, transparent chamber

## Abstract

Many factors play a role in tumour angiogenesis. We observed growing tumour vessels in vivo to study the relationship between blood flow and vascular enlargement. Mammary adenocarcinoma was implanted into Fisher-344 rat with dorsal skin-fold transparent chambers. Vascular growth was observed and recorded on videotape through a microscope for 6 h. Vascular networks were photographed and traced every 30 min to identify changes over time. Tumour sections were stained with Masson's trichrome and anti-Factor VIII-related antigen. Tumour growth was rapid enough for differences to be seen each hour. Vessels with a high blood flow showed an increase in diameter within a few hours and new branches formed from these vessels. In contrast, vessels without an increase in blood flow showed no change in diameter. Vessels within the interstitium surrounding the tumour were lined by endothelium that was positive for anti-Factor VIII-related antigen staining. Vessels in the tumour had extremely rare endothelial cells detectable by Masson's trichrome or anti-Factor VIII-related antigen staining. In conclusion, increased blood flow may cause vascular enlargement and some primitive vessels seem to lack endothelium. *1999 Cancer Research Campaign*


*British Journal of Cancer* (1999) **79**(5/6), 780–786
© 1999 Cancer Research Campaign
Article no. bjoc.1998.0125

### Blood flow influences vascular growth during tumour angiogenesis

**R Nasu, H Kimura, K Akagi, T Murata and Y Tanaka**

Department of Radiology, Kansai Medical University, 10–15, Fumizono-cho, Moriguchi-city, Osaka, 570, Japan

**Summary** Many factors play a role in tumour angiogenesis. We observed growing tumour vessels in vivo to study the relationship between blood flow and vascular enlargement. Mammary adenocarcinoma was implanted into Fisher-344 rat with dorsal skin-fold transparent chambers. Vascular growth was observed and recorded on videotape through a microscope for 6 h. Vascular networks were photographed and traced every 30 min to identify changes over time. Tumour sections were stained with Masson's trichrome and anti-Factor VIII-related antigen. Tumour growth was rapid enough for differences to be seen each hour. Vessels with a high blood flow showed an increase in diameter within a few hours and new branches formed from these vessels. In contrast, vessels without an increase in blood flow showed no change in diameter. Vessels within the interstitium surrounding the tumour were lined by endothelium that was positive for anti-Factor VIII-related antigen staining. Vessels in the tumour had extremely rare endothelial cells detectable by Masson's trichrome or anti-Factor VIII-related antigen staining. In conclusion, increased blood flow may cause vascular enlargement and some primitive vessels seem to lack endothelium.

**Keywords:** neovascularization; experimental tumour; transparent chamber

Many investigators have studied the histologic and morphologic changes associated with the proliferation of new vessels and tumour neovascularization by direct observation using transparent chambers in rats (Yamaura et al, 1971; Hori et al, 1990, 1992, 1993*a*, 1993*b*; Folkman and Klagsbrun 1987; Helmlinger et al, 1997). Recent studies using this technique have revealed a relationship between the branching and formation of tumour vascular networks and arterial blood pressure (Hori et al, 1990; Dewhirst et al, 1992; Patan et al, 1996; Helmlinger et al, 1997). Neovascularization of implanted tumours is rapid for the first 24 h (Hori et al, 1992). Several studies have documented a relationship between vascular growth factors like vascular endothelial growth factor and the formation of a normal vascular network in the presence of ischaemia (Shweiki et al, 1992; Kondo et al, 1993; Gazit et al, 1995; Nagy et al, 1995). The importance of blood flow to neovascularization has long been recognized. Clark et al (1918) noted that vessels with a high blood flow continued to grow, whereas those with little or no blood flow underwent involution. Even vessels adjacent to the area of high blood flow that measured more than 10 μM in diameter underwent involution and were resorbed if local blood flow was decreased or halted. This observation suggested that not only angiogenetic vascular growth factors but also haemodynamic factors play an important role in neovascularization. In the present study, we continuously observed blood vessels in implanted tumours for 6 h under normal physiological conditions using a dorsal flap transparent chamber and a video camera to analyse the relationship between changes of blood flow and tumour vessel diameter during neovascularization.

*Received 3 December 1997*
*Revised 30 June 1998*
*Accepted 2 July 1998*

*Correspondence to:* R Nasu

### MATERIALS AND METHODS

### Transparent chamber

Transparent quartz glass windows (1 mm thick and 1 cm in diameter) with aluminium frames were constructed and applied to rats as described previously (Yamaura et al, 1971).

### Experimental animals and tumour implants

Female Fisher rats aged 7–8 weeks and weighing 130–150 g were obtained from Shimizu Laboratory Animal Center (Shizuoka, Japan) and were used in all experiments. A small piece (0.1 $mm^3$) of mammary carcinoma was implanted under the transparent chamber of each rat. The tumour was rat mammary adenocarcinoma 13762, a carcinogen (dimethylbenz [a] anthracene-induced tumour of female Fisher-344 rat) (American Type Culture Collection Inc., Rocklawn, MD, USA), which forms fold and acini of epithelial tissue and grows to 100 $mm^3$ in about 14 days (Kakeji et al, 1997). The tumour was maintained by subcutaneous transplantation before use in the study. Implanted tumour pieces were held in position using soft agar (0.05%) to all on observation through the window. The subcutaneous tissue within the transparent chamber was 100 μM thick and the implanted tumours formed sheets of cells in this space. We selected one transparent chamber that was growing in a normal physiological condition without inflammation and that was dry in the chamber. The tumour was studied when a mean diameter of 6 mm was reached 25 days after transplantation into the transparent chamber. The animals were kept in a sterile environment and all experiments were performed according to Kansai Medical University's guidelines for animal handling.

### Observation and photography

The rats with transparent chambers were anaesthetized using intraperitoneal nembutal sodium (25 mg/kg) and fixed to a microscope with a specially constructed jig. The body temperature was

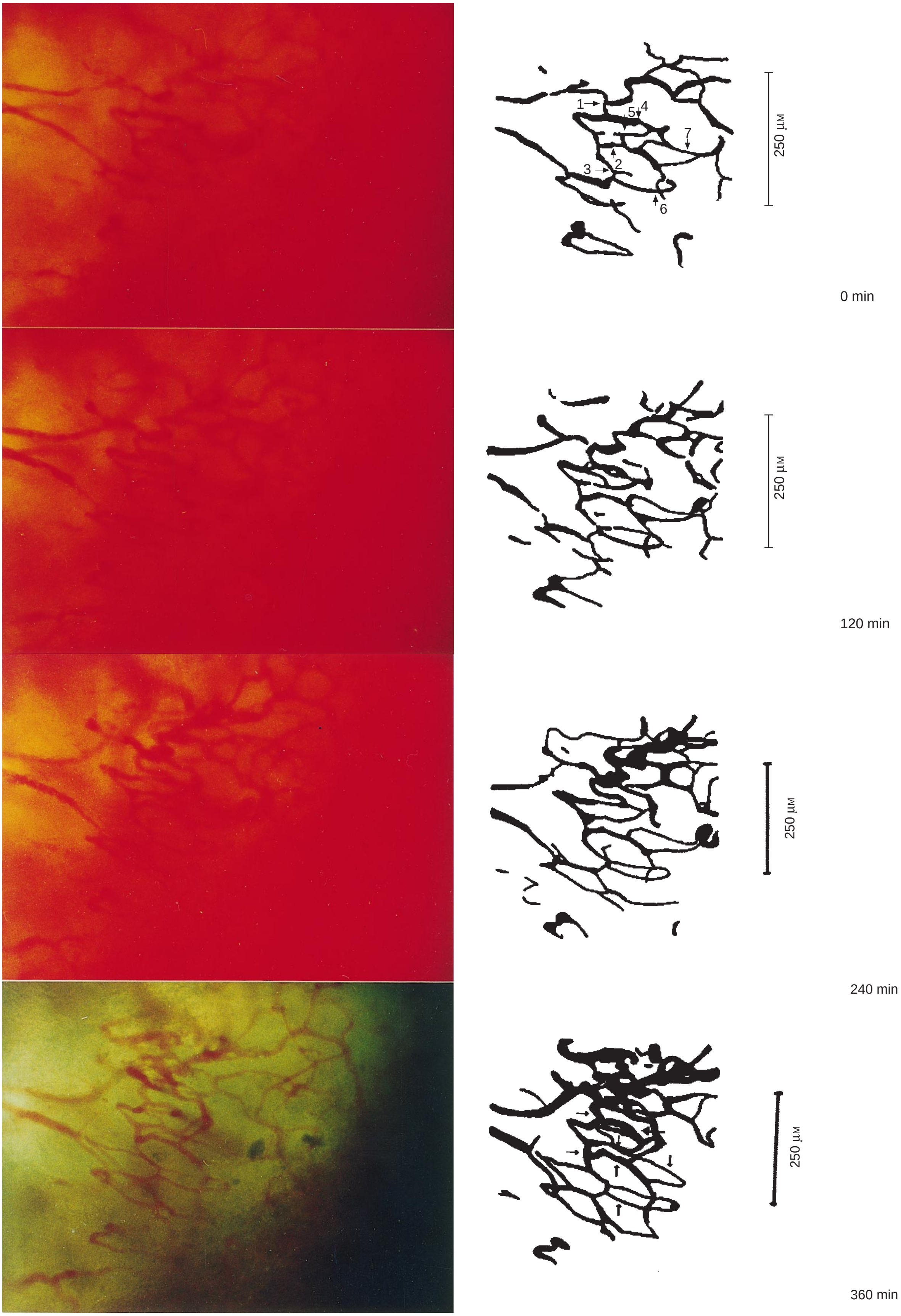

**Figure 1** Photomicrographs obtained every 2 h. The magnification of the objective lens was × 40 and the eyepiece was × 10. Vessel diameters were measured with calipers on the photos

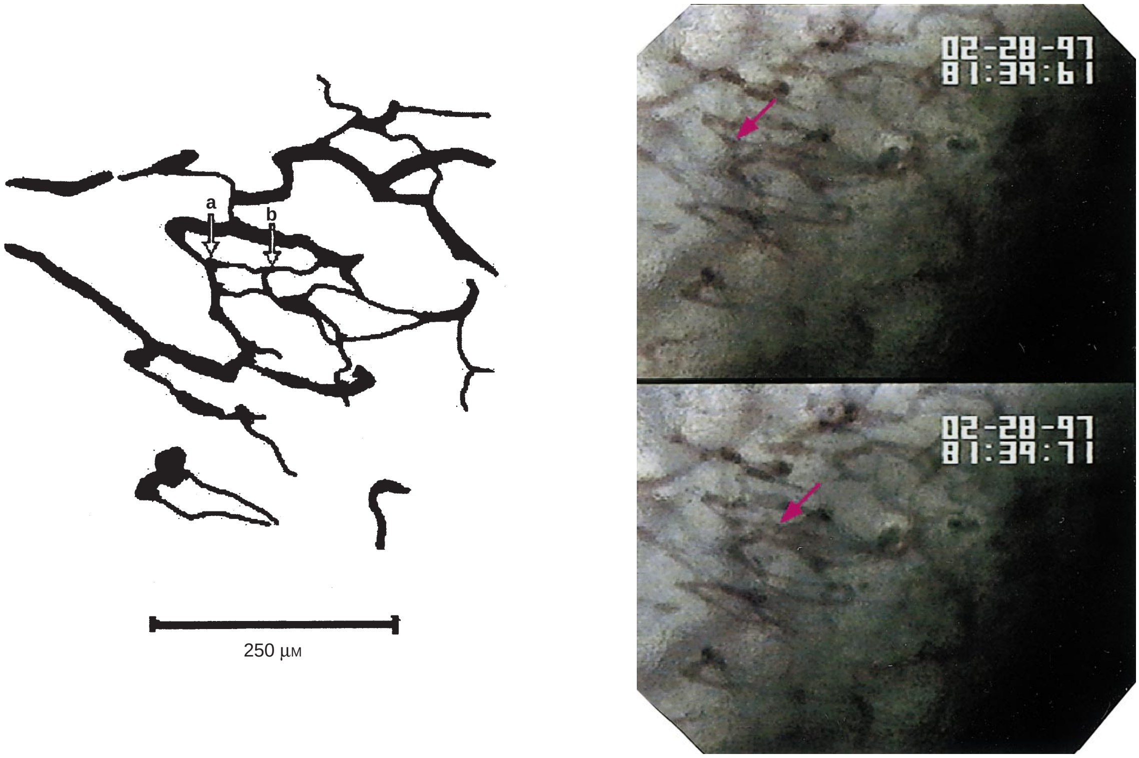

**Figure 2** Video image obtained at the start of observation. The magnification of the objective was × 40 and that of the camera lens was × 16. Positions a (↓) to b (↓) show the distance an erythrocyte moved in 0.1 s maintained for 6 h using a heating pad while the tumours were observed and tumour growth was recorded using a video camera (Victor Japan, Tokyo, Japan) attached to the microscope (Optiphoto-2, Nikon, Japan). Photomicrographs were obtained every hour (magnification × 640). Our observations were done under nembutal sodium anaesthesia, which can affect the blood pressure and heart rate, but we observed the same rat for 6 h so we could obtain stable data.

### Measurement of changes in vessel diameter and blood flow

### *Measurement of vessel diameter*

The diameters of the seven vessels shown in Figure 1, ranging from 7.14 to 16.07 μM, were measured with calipers on the photomicrographs obtained every hour. Measurements were corrected by the magnifying ratio to obtain the actual diameters.

### *Measurement of blood flow*

Figure 2 shows a typical video image obtained at the start of the observation period. The distance (points a–b) that an erythrocyte travelled along the vessel in 0.1 s was measured with calipers and corrected by the magnifying ratio to obtain the blood flow rate (μM/0.1 s). To minimize errors, the blood flow rate was measured 10 times in the same vessel, and the mean value was calculated.

Changes in the diameter and blood flow of the seven vessels were determined every hour for 6 h. The rate of change of each parameter was calculated as a ratio by setting the value obtained at the start of observation as 1.00. The seven tumour vessels were assigned to groups showing an increase in diameter or no change in diameter (expanding and non-expanding groups, respectively). Only a few vessels could be used to measure the blood flow and diameter over 6 h accurately, because many new branch vessels formed and the direction of blood flow often changed during angiogenesis. In addition, growth of the tumour tissue led to decreasing visibility, so it was difficult to identify the same vessels for 6 h. As a result, we could only measure the blood flow and diameter of seven vessels.

**Table 1** The changes of vessel diameter (μM)

| | Number of vessels | | | | | | |
|---|---|---|---|---|---|---|---|
| | Expanding | | | Non-expanding | | | |
| Time (min) | 1 | 2 | 3 | 4 | 5 | 6 | 7 |
| 0 | 8.93 | 10.71 | 7.14 | 16.07 | 7.14 | 7.14 | 7.14 |
| 60 | 8.93 | 17.86 | 13.40 | 22.33 | 8.93 | 7.14 | 7.14 |
| 120 | 10.71 | 13.40 | 16.70 | 10.71 | 8.93 | 7.14 | 4.47 |
| 180 | 8.93 | 17.86 | 8.93 | 10.71 | 7.14 | 7.14 | 7.14 |
| 240 | 13.40 | 13.40 | 8.93 | 10.71 | 8.93 | 6.25 | 4.47 |
| 300 | 17.86 | 17.86 | 10.71 | 10.71 | 8.93 | 7.14 | 4.47 |
| 360 | 13.40 | 13.40 | 16.07 | 8.93 | 8.93 | 7.14 | 4.47 |

Hourly changes of vascular diameters in the expanding (nos 1, 2 and 3) and non-expanding (nos 4, 5, 6 and 7) groups.

### Staining of tumour sections

After observing the blood vessels for 6 h, the rats were killed and the tumours were removed and fixed in formalin. Tumour sections

© Cancer Research Campaign 1999

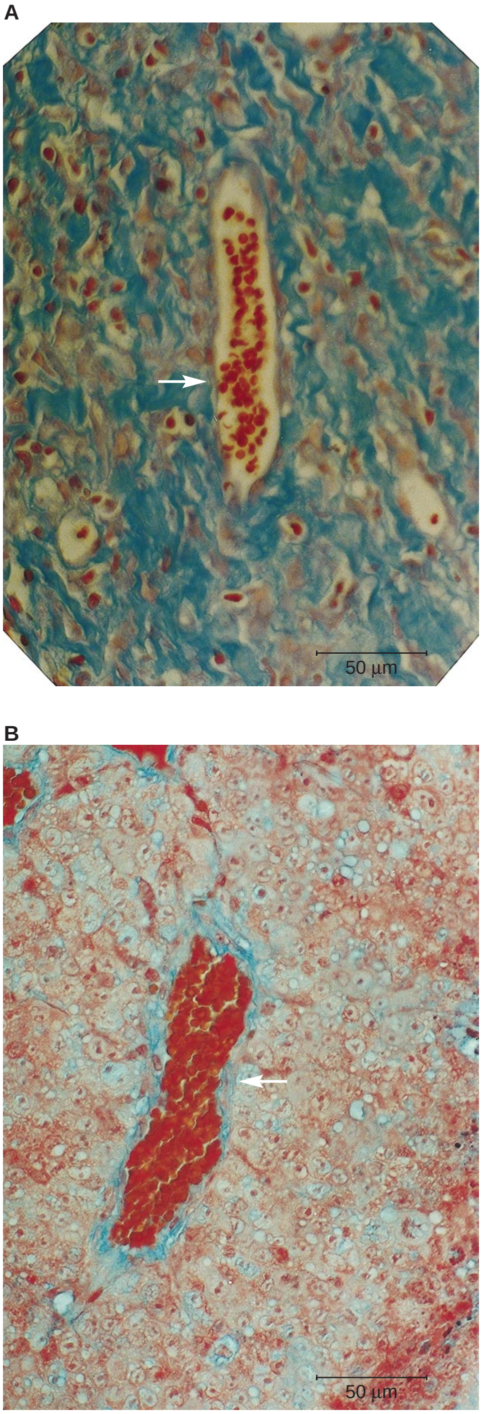

**Figure 3** Histology of tumours grown in rat dorsal flap transparent chambers. (**A**) Masson's trichrome stain demonstrates fibrous tissue adjacent to the non-tumour vessels (↓). (**B**) There is little fibrous tissue adjacent to vessels of similar diameter within the tumour (↓). Part of the tumour vessel wall has no endothelial cells or fibrous tissue were cut and stained with Masson's trichrome stain and a rabbit antibody for Factor VIII-related antigen (Tanner, Kobe, Japan) (Otsuki et al, 1990). Then histological examination was done at a magnification of × 400 (Figure 3).

**Table 2** The changes of blood flow in expanding and non-expanding groups (µM/0.1 s)

| | Number of vessels | | | | | | |
|---|---|---|---|---|---|---|---|
| | Expanding | | | Non-expanding | | | |
| **Time (min)** | **1** | **2** | **3** | **4** | **5** | **6** | **7** |
| 0 | 25.02 | 30.86 | 31.97 | 41.14 | 60.88 | 31.69 | 30.02 |
| 60 | 22.24 | 32.53 | 37.81 | 29.75 | 43.09 | 30.30 | 30.02 |
| 120 | 43.09 | 30.58 | 36.70 | 43.92 | 38.92 | 31.40 | 31.40 |
| 180 | 47.26 | 38.36 | 41.42 | 45.59 | 41.70 | 32.53 | 31.40 |
| 240 | 48.37 | 43.65 | 44.76 | 41.14 | 47.82 | 32.25 | 35.58 |
| 300 | 45.59 | 56.71 | 47.82 | 48.09 | 42.26 | 40.87 | 39.20 |
| 360 | 34.47 | 48.93 | 43.65 | 51.15 | 61.99 | 42.53 | 35.58 |

### RESULTS

Table 1 shows changes of diameter over time in the two groups of vessels. Vessels in the expanding group showed an increase in vascular diameter over the 6 h of observation. Vessels in the non-expanding group either contracted or did not change in size. The mean rate of change was 1.67 for the expanding group and 0.97 for the non-expanding group (Figure 4).

Table 2 shows the changes in blood flow rates over time for the two groups. The blood flow rate increased in both groups, but the change was more marked in the expanding group. The mean rate of change at 6 h was 1.442 for the expanding group and 1.197 for the non-expanding group (Figure 5).

Figures 3 and 6 show tumour growing in a transparent chamber. Masson's trichrome-stained sections revealed fibrous tissue adjacent to the vessels in the areas surrounding the tumour (Figure 3A), but little fibrous tissue adjacent to vessels of a similar diameter within the tumour (Figure 3B). The anti-Factor VIII-related antigen-stained sections showed a uniform distribution of endothelial cell nuclei in the vessels within the interstitium surrounding the tumour (Figure 6A), and a non-uniform distribution of endothelial cell nuclei in the tumour vessels (Figure 6B). Vessels in the tumour had extremely rare endothelial cells.

### DISCUSSION

Although there are a few reports directly observing the growth of tumours and the formation of tumour vessel networks (Ide et al, 1939; Algire, 1943), the effects of administering vasoactive substances and angiogenesis inhibitors on neovascularization have been studied in the past few years (Dewhirst et al, 1992; Hori et al, 1992, 1993*a*). Neovascularization of tumours has been assessed in terms of arterial branching, the lengthening of blood vessels and the effect of arterial blood pressure (Dewhirst, 1992; Hori et al, 1992, 1993*a*).

It has been suggested that tumour neovascularization is stimulated by the death of tumour cells, because cells surrounding the tissue release vascular growth factors. These angiogenetic factors stimulate vascular endothelial cells to secrete proteolytic enzymes including plasminogen activator and collagenase. These enzymes break down the basement membrane to produce small defects in the vessel wall, which endothelial cells can then cross to form new vessels (Patan et al, 1996). Folkman and Klagsbrun (1987) reported that endothelial cells become organized into tubular structures (capillary loops) and form anastomoses between

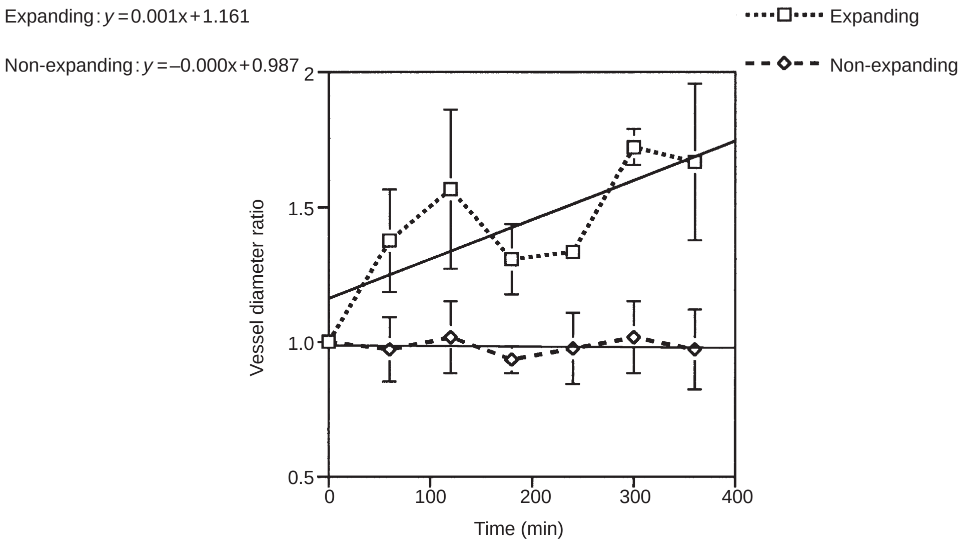

**Figure 4** The time-course of vessel diameter changes in the expanding and non-expanding groups

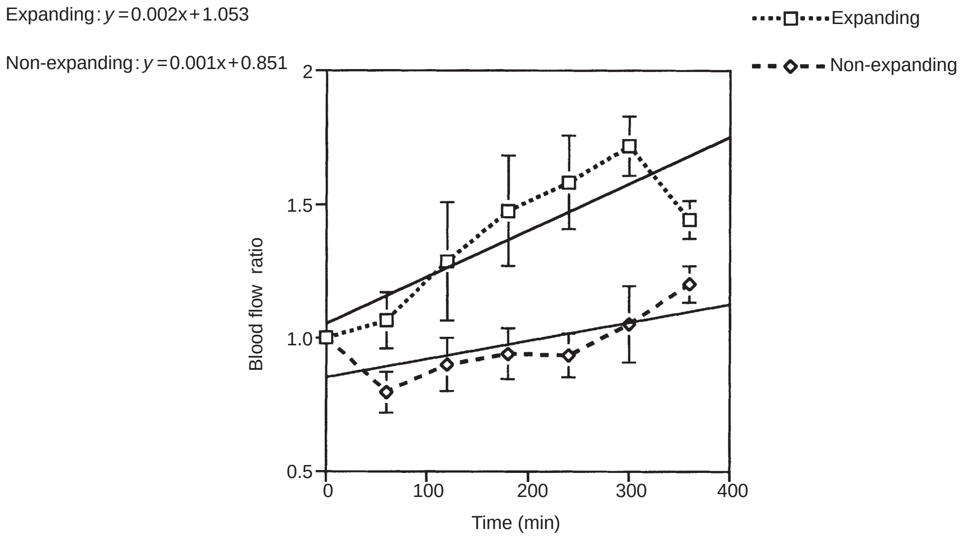

**Figure 5** The time-course of mean blood flow rates in the two groups themselves and elements of the host vasculature during the establishment of a primitive tumour circulation. However, Baillie et al (1995) have found that tissues with pre-existing blood vessels formed tumour vascular networks by a process that differs from Folkman's model.

In the present experiment, we observed no sprouting of new blood vessels. In all tumours, primitive blood flow of both red and white blood cells was seen between individual tumour cells. Some of the vessels with increasing flow rates became dedicated tumour blood vessels, whereas others stopped growing depending on the pressure gradient. In the latter case, primitive blood vessel flow was small and eventually ceased.

Clark (1918) studied the normal vascular network in tadpole tails and provided evidence supporting Thoma's theory:

1. an increase or decrease in vessel size is regulated by the rate of blood flow
2. an increase or decrease in vessel length is governed by the tension exerted on the vessel wall in a longitudinal direction by the surrounding tissues
3. an increase or decrease in vessel wall thickness is dependent on blood pressure
4. formation of new capillaries depends upon the increase in pressure of the capillary (Thoma, 1893; Wu et al, 1993).

Our present findings agree with those of Clark (1918). Implantation of a tumour adjacent to a normal vessel (within 100 μM) led to tumour growth. Two types of vessels appeared to form the tumour vascular network: some normal host vessels became tumour vessels, and other tumour vessels grew de novo. It is well known that microhaemodynamics of tumour vessels varies at different locations in a tumour. We investigated vessels that were growing rapidly at the periphery of the tumour. We had much data about tumour neovascularization using the transparent

© Cancer Research Campaign 1999

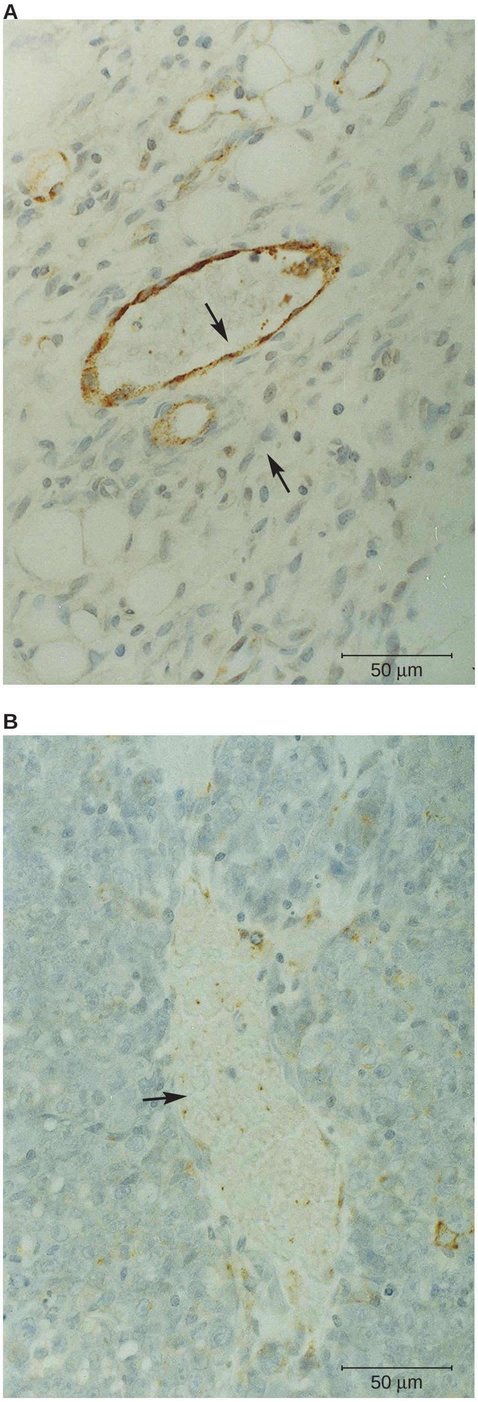

**Figure 6** (**A**) Staining with anti-Factor VIII-related antigen shows a uniform distribution of endothelial cell nuclei in the non-tumour vessels (↓). (**B**) In contrast, there is a non-uniform distribution of endothelial cell nuclei in the tumour vessels (↓)

chamber. We did not analyse qualitative findings of other tumour angiogenesis over 6 h, but observations showed the same tendency. It was possible to analyse changes of only a few tumour vessels. In the light microscopic field, we could find vessels that showed increasing or decreasing blood flow and diameter. The tumour vascular network appeared to form as follows.

First, primitive blood vessels that coursed through intercellular sinusoids and between tumour cells were formed through which only one or two red blood cells could pass abreast. These primitive blood vessels might then form loop vessels (10–20 µM), with a diameter large enough for three to five red blood cells to pass. Such vessels showed only intermittent flow. If blood flow in such a vessel became obstructed, the vessel disappeared. However, if blood flow in the loop vessel increased, the loop became larger. An increase in the number of loops subsequently led to an increase in loop diameter. Histologic examination of these loop vessels did not reveal endothelial cells. Finally, the loop vessels enlarged and joined with adjacent vessels to form a new flow path. Loop vessels with increasing blood flow showed an increase in diameter (35–50 µM) and became tumour vessels. In many cases, the walls of these tumour vessels were not uniformly lined by endothelium (Figure 6). It has been reported that cells other than endothelial cells are involved in forming the walls of tumour vessels (Baillie et al, 1995). In our study, Masson's trichrome-stained sections revealed little fibrous tissue adjacent to blood vessels near the interstitial vascular lumens that surrounded the tumour, and some vessels had no lining of endothelial cells (Figure 3).

In conclusion, these findings suggest that the continued growth of tumour blood vessels depends more on local haemodynamics than on vascular growth factors.

### REFERENCES

Algire GH (1943) An adaptat:on of the transparent chamber technique to the mouse. *J Natl Cancer Inst* **4**: 1–11

Baillie CT, Winslet MC and Bradley NJ (1995) Tumour vasculature a potential therapeutic target. *Br J Cancer* **72**: 257–267

Clark ER (1918) Studies on the growth of blood vessels in the tail of the frog larva by observation and experiment and on the living animal. *Am J Anat* **23**: 37–88

Dewhirst MW, Vinuya RZ, Ong ET, Klitzman B, Rosner G, Secomb TW and Gross JF (1992) Effects of bradykinin on the hemodynamics of tumor and granulating normal tissue microvasculature. *Radiat Res* **130**: 345–354

Folkman J and Klagsbrun M (1987) Angiogenic factors. *Science* **235**: 422–447

Gazit Y, Berk DA, Leunig M, Baxter LT and Jain RK (1995) Scale-invariant behavior and vascular network formation in normal and tumor tissue. *Phys Rev Lett* **75**: 2428–2431

Helmlinger G, Yuan F, Dellian M and Jain RK (1997) Interstitial pH and $pO_2$ gradients in solid tumors in vivo: high-resolution measurements reveal a lack of correlation. *Nature Med* **3**: 177–182

Hori K, Suzuki M, Tanda S and Saito S (1990) In vivo analysis of tumor vascularization in the rat. *Jpn J Cancer Res* **81**: 279–288

Hori K, Suzuki M, Saito S, Tanda S, Shinozaki M and Zhang Q (1992) Circardian variation of tumor blood flow in rat subcutaneous tumors and its alteration by angiotensin II-induced hypertension. *Cancer Res* **52**: 912–916

Hori K, Zhang Q-H, Saito S, Tanda S, Li H and Suzuki M (1993*a*) Microvascular mechanisms of change in tumor blood flow due to angiotensin II, epinephrine, and methoxamine: A functional morphometric study. *Cancer Res* **53**: 5528–5534

Hori K, Suzuki M, Tanda S (1993*b*) Functional characterization of developing tumor vascular system and drug delivery (review). *Int J Oncol* **2**: 289–296

Ide AG, Baker NH and Warren SL (1939) Vascularization of the Brown–Pearce rabbit epithelioma transparent as seen in the transparent ear chamber. *Am J Roentgenol* **42**: 891–899

Kakeji Y, Maehara Y, Ikebe M and Teicher BA (1997) Dynamics of tumor oxygenation, CD31 staining and transforming growth factor-β levels after treatment with radiation or cyclophosphamide in the rat 13762 mammary carcinoma. *Int J Radiation Oncology Biol Phys* **37**: 1115–1123

Kondo S, Asano M and Suzuki H (1993) Significance of vascular endothelial growth factor/vascular permeability factor for solid tumor growth, and its inhibition by the antibody. *Biochem Biophys Res Commun* **194**: 1234–1241

Nagy JA, Morgan ES, Herzberg KT, Manseau EJ, Dvorak AM and Dvorak HF (1995) Pathogenesis of ascites tumor growth: angiogenesis, vascular remodeling and stroma formation in the peritoneal lining. *Cancer Res* **55**: 376–385

*© Cancer Research Campaign 1999*

Otsuki Y, Kubo H and Magari S (1990) Immunohistochemical differentiation between lymphatic vessels and blood vessels use of anti basement membrane antibodies and anti-factor VIII related antigen. *Arch Histol Cytol* **53**: 95–105
Patan S, Munn LL and Jain RK (1996) Intussusceptive microvascular growth in a human colon adenocarcinoma xenograft: a novel mechanism of tumor angiogenesis. *Microvascular Res* **51**: 260–272
Shweiki D, Itin A, Soffer D and Keshet E (1992) Vascular endothelial growth factor induced by hypoxia may mediate hypoxia-initiated angiogenesis. *Nature* **359**: 843–845
Thoma R (1893) *Untersuchungen uber die Histogenasa und Histomechanik des Gefassystems*. Enkeverlag: Stuttgart
Wu NZ, Da D, Rudoll TL, Needham D, Whorton AR and Dewhirst MW (1993) Increased microvascular permeability contributes to preferential accumulation of stealth liposomes in tumor tissue. *Cancer Res* **53**: 3765–3770
Yamaura H, Suzuki M and Sato H (1971) Transparent chamber in the rat skin for studies on microcirculation in cancer tissue. *GANN* **62**: 177–185

 *© Cancer Research Campaign 1999*
